# A Phase 2 Randomised Clinical Trial Assessing the Tolerability of Two Different Ratios of Medicinal Cannabis in Patients With High Grade Gliomas

**DOI:** 10.3389/fonc.2021.649555

**Published:** 2021-05-21

**Authors:** Janet Schloss, Judith Lacey, Justin Sinclair, Amie Steel, Michael Sughrue, David Sibbritt, Charles Teo

**Affiliations:** ^1^ National Centre for Naturopathic Medicine, Southern Cross University, Lismore, NSW, Australia; ^2^ Office of Research, Endeavour College of Natural Health, Brisbane, QLD, Australia; ^3^ Australian Research Centre in Complementary and Integrative Medicine, University of Technology, Sydney, NSW, Australia; ^4^ National Institute of Complementary Medicine (NICM) Health Research Institute, Western Sydney University, Sydney, NSW, Australia; ^5^ Supportive Care, Chris O’Brien Lifehouse Cancer Hospital, Sydney, NSW, Australia; ^6^ Clinical School of Medicine, University of Sydney, Sydney, NSW, Australia; ^7^ Prince of Wales Private Hospital, Centre for Minimally Invasive Neurosurgery, Sydney, NSW, Australia

**Keywords:** glioblastoma multiforme (GBM), cannabis (marijuana), medicinal marijuana, high-grade glioma (HGG), tolerability, quality of life

## Abstract

**Background:**

Cannabis for cancer is very topical and, given the use of illicit cannabis preparations used in this vulnerable population, research investigating standardised, quality-assured medicinal cannabis is critical to inform clinicians and assist patient safety.

**Methods:**

A randomized trial involving adult patients diagnosed with a high-grade glioma, no history of substance abuse, liver or kidney damage or myocardial infarction were eligible for inclusion in a tolerability study on two different ratios of medicinal cannabis. Baseline screening of brain morphology, blood pathology, functional status, and cognition was conducted. A retrospective control group was used for comparison for secondary outcomes.

**Results:**

Participants (n=88) were on average 53.3 years old. A paired t-test assessed the Functional Assessment of Cancer Therapy for Brain Cancer (FACT-Br) between groups from baseline to week 12 found that the 1:1 ratio favoured both physical (p=0.025) and functional (p=0.014) capacity and improved sleep (p=0.009). Analysis of changes from baseline to week 12 also found 11% of 61 participants had a reduction in disease, 34% were stable, 16% had slight enhancement, and 10% had progressive disease. No serious adverse events occurred. Side effects included dry mouth, tiredness at night, dizziness, drowsiness.

**Conclusion:**

This study demonstrated that a single nightly dose of THC-containing medicinal cannabis was safe, had no serious adverse effects and was well tolerated in patients. Medicinal cannabis significantly improved sleep, functional wellbeing, and quality of life.

**Clinical Trial Registration****:**

Australian New Zealand Clinical Trials Registry (ANZCTR) http://www.anzctr.org.au/Trial/Registration/TrialReview.aspx?id=373556&isReview=true, identifier ACTRN12617001287325.

## Introduction

In recent years, the medicinal properties of the *Cannabis* genus have received increased attention from researchers around the world alongside regulatory changes in many jurisdictions affecting clinician and public access to cannabis. Current evidence suggests medicinal cannabis (MC) – defined here as cannabis being used for medical purposes - may inhibit chemotherapy-induced nausea and vomiting, stimulate appetite, reduce pain, and decrease inflammation and cancer cell proliferation and survival (1, 2). Based on these effects, numerous medical conditions and health complaints have been identified to potentially benefit from the clinical application of cannabis. These include cancer-related refractory nausea, anorexia and pain, chronic pain conditions, sleep disturbances, refractory epilepsy, multiple sclerosis, muscle spasms and seizures, with studies continuing in Parkinson’s disease, hypoxic brain injury, movement disorders, Alzheimer’s disease among others (2–4).

The pharmacological properties of cannabis are primarily linked to the activity of cannabinoids, a group of terpenophenolic constituents (5). Currently, over 140 cannabinoids have been identified (6, 7). The compound which has been most widely studied is delta-9– tetrahydrocannabinol (THC) while the other main cannabinoid compound with reported beneficial properties is cannabidiol (CBD) (8). THC has diverse therapeutic actions (9–23). It is a partial agonist of cannabinoid receptor type 1 and 2 (CB_1_ and CB_2_) (24, 25), expressing similarity to the endogenous cannabinoid anandamide (23, 26–28). THC is the main intoxicating phytochemical, however its main active metabolite after absorption is 11-hydroxy-Δ^9^-tetrahydroxannabinol (11-OH-THC), which has higher therapeutic potency (24, 29) and blood brain barrier (BBB) permeability. CBD is an antagonist of cannabinoid receptor agonists, and may offset the psychoactivity of THC when co-administered - reducing symptoms such as paranoia, dysphoria and anxiety (30–35) while also potentiating cannabis tolerability (36, 37). CBD is anti-inflammatory (38), but also exhibits other diverse effects including neuroprotective (39), anticonvulsive (40), antipsychotic (27, 41–43), anxiolytic (44), antidepressant (45), hypnotic, sedative, anticancer (46–51), analgesic and antiemetic activity (27). The anticancer properties of cannabinoids were first proposed in 1975 when Munson et al. (52) demonstrated THC and the related compound delta 8-THC as well as cannabinol (CBN) (but not CBD) reduced primary cancer growth through *in vitro* and *in vivo* lung adenocarcinoma growth murine models. Recent reviews have concluded that a large number of cannabinoid compounds have been discovered, developed, and used for cancer either alone or in combination with standard anti-cancer strategies. From these reviews, MC could become a therapy of choice in contemporary oncology (53) however, the tolerability of high dose THC has not been conducted in this brain cancer cohort.

Gliomas are the most common primary intracranial tumour of the central nervous system, accounting for one third of all brain tumours (54). Glioblastoma multiforme (GBM) is the most aggressive form of human brain tumour and are normally incurable due to the high recurrence rate, even after total resection (55, 56), with a median survival rate of approximately one year (57). Treatment of GBMs and high-grade gliomas such as anaplastic astrocytoma grade III (AA3)’s is challenged by their location, aggressive biological behaviour, diffuse infiltrative growth and low survival rate (58). Standard GBM and high-grade glioma (HGG) treatment involves surgical resection followed by adjuvant radiation therapy and chemotherapy (55). Non-clinical studies which have examined the effects of MC on high grade gliomas have been summarised in two systematic reviews conducted by Rocha et al. (59) and Rodriguez-Almaraz et al. (60). The 2014 review identified several key findings including details of the cellular and molecular mechanisms through which cannabinoids impact high grade gliomas. It reported evidence that CBD inhibits tumour angiogenesis, decreases the risk of metastasis and induces tumour cell apoptosis (59). The 2020 review found that there was limited moderate-quality evidence that supports the use of cannabinoids as an adjuvant to the standard care in the treatment of brain tumours and suggests that further prospective trials be conducted (60).

Clinical trials investigating the efficacy of MC for cancer have mainly examined cancer-related symptoms with limited trials focusing on cancer. One 2006 pilot trial involving patients with recurrent GBM (61) examined intra-tumour administration of THC on nine patients and reported a good safety profile (no significant changes in physical, neurological, biochemical or haematological parameters were noted) and possible anti-proliferative activity on the tumour cells (61).

The tolerability of high dose THC in different patient groups and the impact of MC on standard treatment efficacy is currently unknown. Given the preliminary research examining MC in patients with GBM (61), and the poor prognosis associated with this disease, we designed a trial to assess the tolerability of THC containing cannabis products.

## Patients and Methods

### Primary Objective

To investigate the tolerability of two different ratios of oral medicinal cannabis oil in patients who have been diagnosed with high grade gliomas (GBM or astrocytoma AA3r) as an adjunct to standard treatment.

### Study Design

A single-centre Phase II double-blind randomised clinical trial assessing the tolerability of two different cannabinoid ratios of oral MC oil on patients with high-grade gliomas was conducted.

### Participants

The study population for this trial were patients diagnosed with a recurrent or inoperable high-grade glioma (GBM or astrocytoma AA3r). They were aged 18 years or older, willing to participate in a clinical trial using MC and had no history of substance abuse. Each patient was assessed for European Cooperative Oncology Group score for functional performance status (ECOG) and screened for cognitive function *via* the general practice cognitive screening test (GPCOG) before being enrolled. Baseline blood tests were taken to ascertain cannabis metabolite levels prior to enrolment. They were excluded if they were pregnant or breast feeding, had severe cognitive impairment, severe mental illness, non-English speaking, had a past history of major substance abuse, had an adverse event from past cannabis use, severe liver or kidney disease or had a past history of myocardial infarctions. In addition, if participants were required to have another craniotomy while participating in the trial, they were excluded from the study. The study was conducted at the Centre for Minimally Invasive Neurosurgery, Prince of Wales Private Hospital, NSW Australia from November 2018 to December 2019. The participants diagnosis was confirmed by one of the participants clinicians or if not possible, the doctors at the Centre for Minimally Invasive Neurosurgery. The confirmation of diagnosis was based on magnetic resonance imagining (MRI) discs and histopathology results from biopsy and/or surgery. No molecular markers were used so a range of genetic variations including 06-Methylguanine-DNA Methyltransferase (MGMT) promoter methylation, deletions of genes and mutations (IDH1, IDH 2) were included. As a pilot trial to ascertain any specific information, it was decided that a pragmatic approach be used for this trial to inform the next phase.

The advisory board and all investigators approved the study protocol. All patients provided written informed consent before study interventions were initiated. All researchers carried out the clinical trial in accordance with The Code of Ethics of the World Medical Association (Declaration of Helsinki) and all possess a current Good Clinical Practice (GCP) certificate.

### Investigation Products

The investigational products were sourced through Bioceuticals Pty Ltd from Whistler Medical Marijuana Corporation (WMMC) and consisted of two standardised and quality-assured specially formulated oil-based organic whole plant extracts of cannabis based on a 1:1 and 4:1 ratio of THC : CBD (1:1 THC 4.6mg/ml:CBD 4.8mg/ml and 4:1 THC 15mg/ml:CBD 3.8mg/ml) compliant with Therapeutic Goods Order # 93 (62). The investigational products were specifically made to conform with Good Manufacturing Practice (GMP) and were tested for quality to comply with Therapeutics Goods Administration (TGA), the Australian Federal regulator, requirements. Patients were given a single dose at night before bed that was titrated up to tolerance starting at 0.20ml (maximum 5ml in one dose).

### Outcomes

The primary outcome of this trial was assessed by side effects and the Functional Assessment of Cancer Therapy- Brain (FACT-Br) (63) from baseline to 12 weeks in addition to patient tolerability of the oils *via* a participant diary. The FACT-Br is a commonly used quality of life (QoL)questionnaire for cancer related issues with questions specific to patients with brain cancer (63). Secondary outcomes included treatment-related toxicity, blood safety markers, dose response and tumour growth over 12 weeks. The blood cannabinoid metabolites and endocannabinoid level analysis will also be conducted and presented at a later date.

### Randomisation

The randomisation sequence was conducted by the principal investigator and the allocation was only known by one Bioceuticals staff member responsible for coordinating product labelling. All researchers, pharmacists, doctors, patients, and staff were blinded to the allocation until after all analysis was completed. The reason for blinding the interventions, even though one was not a placebo, is because they both contained THC and the researchers wanted to minimize the possible effects of experimenter bias. A researcher being able to identify the intervention could potentially result in the researchers unknowingly influencing the results during the administration of the trial, data collection or the analysis.

The randomisation sequence was conducted using Research Randomizer: https://www.randomizer.org/. The randomisation was conducted for two sets using 46 participants. Allocation was *via* an on-line data management system, Clinical Studio, with the allocation not known until the person had been entered as enrolled into the system.

### Drug Administration

Each intervention did not have a pharmacological name on the product. Both interventions were displayed in plain bottles with trial labels on them. They were labelled with either Arm A or B to distinguish between the groups. All investigator products were kept in a Schedule 8 facility in the private hospital pharmacy and were dispensed upon presentation of a trial script. The research nurse explained the administration of the drugs, titration information, dosing regimen, and potential side effects to each participant. Upon dispensing the drug, the trained pharmacist also explained the administration, dose, and potential side effects. The trial nurse contacted the participants every two days to ensure any adverse effects, dosage and increase or decrease of dosage until tolerated dose was reached.

### Procedures

Treatment was conducted for 12 weeks in conjunction with standard treatment. Follow-up appointments occurred at 4, 8 and 12 weeks. A baseline and 12-week MRI scan were conducted and analysed using the RANO criteria (64) by a single centre radiologist at iMed radiology. Blood pathology was taken at each time point and toxicities were graded using the National Cancer Institute Common Terminology Criteria for Adverse events (NCI-CTC: version 4.0) (65). The patients completed the FACT-Br (63) at each time point.

The MRIs were conducted with contrast in a 3T Phillips Dual Weighted-MRI scanner using single-shot spinecho EPI, axial and free breathing techniques at the IMed Prince of Wales Private Hospital. The blood pathology was collected and analysed by New South Wales (NSW) Health Pathology at baseline, 4 weeks, 8 weeks, and 12 weeks. Each blood test included electrolytes, liver function, kidney function, basal serology, C reactive protein, full blood count and if the participant was on phenytoin or carbamazepine, they were also tested each collection.

### Retrospective Data Control Group

A retrospective control group acted as the historical control. The selection of the historical controls was matched for gender, age, disease state, MRI scans (12 weeks apart) and treatment. All historical controls were screened for no medicinal cannabis use during this time. This will be verified by what the doctor documented for other use and that it didn’t include medicinal cannabis.

A retrospective group was chosen to compliment the tolerability study of two different ratios of medicinal cannabis. Due to the absence of a prospective control group and comparing one ratio to another, the researchers decided it would be beneficial to have a comparison, hence the retrospective group was chosen.

The historical controls were patients of Dr Teo’s and his fellows. Medical records were accessed at the Centre of Minimally Invasive Neurosurgery, at the Prince of Wales Hospital, Randwick. The research nurse had access to the medical records at the Centre, and completed the Retrospective Data Collection Form (RDCF). No information on the form was identifiable and the medical records were not taken from the Centre.

### Sample Size Calculation

No published study was found to be similar to this trial in terms of oral administration of the intervention, outcome measure, dosage of the intervention or time frame. Therefore, the sample size calculation was based on a trial conducted to examine high grade gliomas that used the standard deviation for the FACT-Br which is the primary outcome for this trial (66). To detect a difference between the intervention group and the control group of a change in FACT-Br (SD=8) with 80% power for 5% significance, we were required to recruit 82 participants (41 per arm). This allowed for a 20% attrition rate.

### Statistical Analysis

Stata Version 14 was utilised to analyse all data using an intention-to-treat (ITT) basis. For the primary outcome measure, paired T tests and longitudinal mixed model analysis including mixed methods and generalized estimating equation [GEE] was used, with adjustments for potential confounders, including age and gender. Measures of safety and adverse events were analysed using the GEE to assess differences over time. Comparison to the retrospective historical data was conducted using unpaired t-tests. Evaluation of tumour growth was assessed using the RANO criteria from baseline to 12 weeks comparing the two arms. Statistical significance was set at p<0.05.

### Ethics

The trial was approved by three human research ethics committees (HREC): South Eastern Sydney Local Health District HREC: 18/028, University of Technology Sydney HREC: ETH 18-2761 and Endeavour College of Natural Medicine HREC: 20180821.

## Results

**Figure 1:**
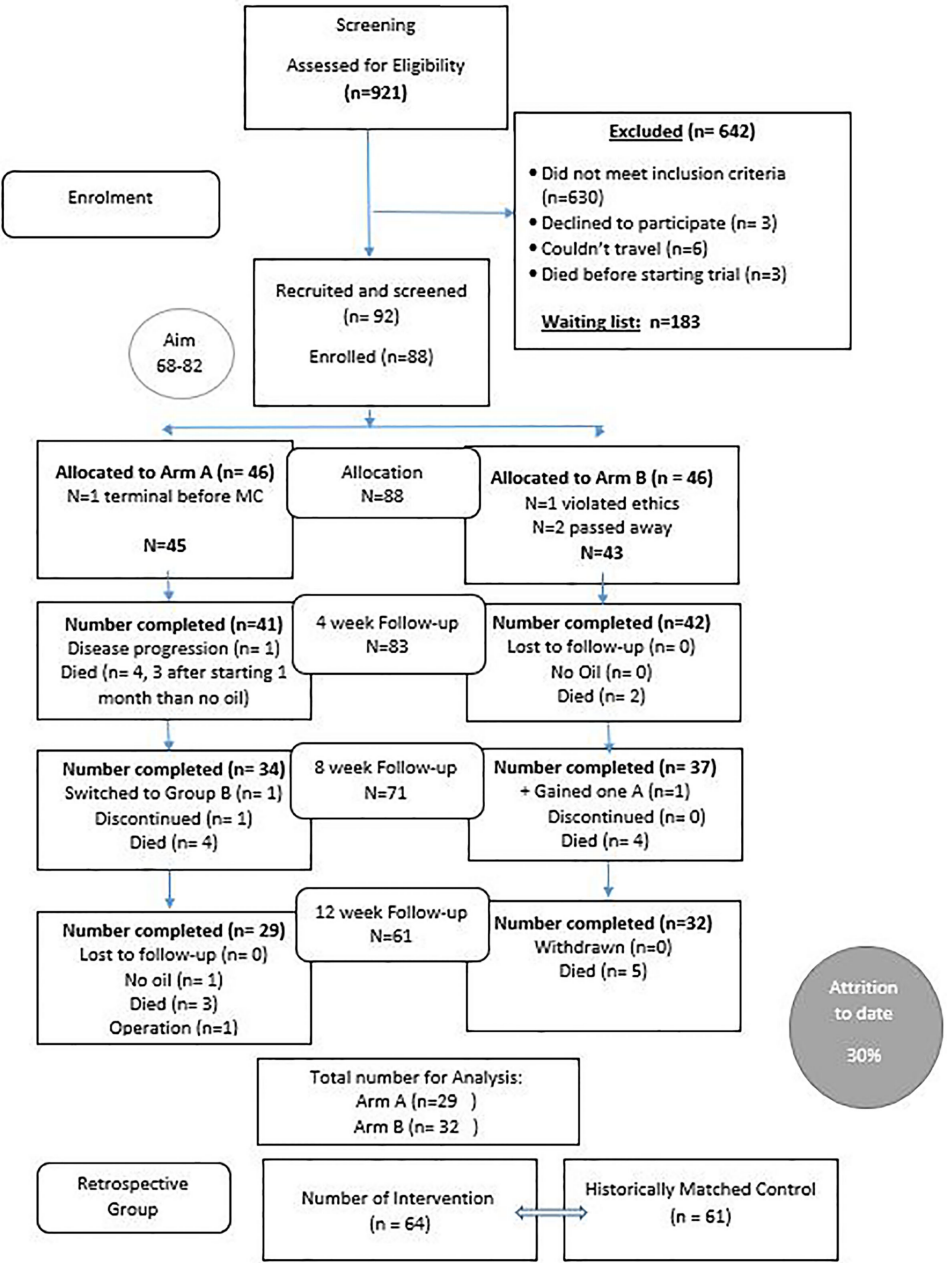
Consort diagram for the Cannabis and GBM trial.

Nine hundred and twenty-one patients were initially screened for this trial due to a media release. Of those, 642 were excluded due to not meeting the inclusion criteria (many had brain metastasis or other brain cancers: see **Figure 1**) and 92 were recruited from around Australia and further screened by the study medical doctor (MS) for liver and kidney function and review of MRI results in addition to the research nurse screening for cognition. Eight-eight (88) participants were enrolled with 61 completing the 12-week intervention. As anticipated, an attrition rate of 30% occurred with 27 (29%) of the 92 dying before or during the trial. (see **Figure 1**).

### Patient Characteristics

Ninety-two (92) patients were recruited and screened with 88 participants enrolled and 83 completing at least 4 weeks of the intervention (1:1 ratio n=41; 4:1 ratio n=42). The majority were Caucasian with a similar breakdown between females and males (1:1 female 56%, males 44%; 4.1 females 43%, males 57%). The average age of both groups was 53.3 years with an average BMI of 25.7. Thirty (n=30) participants were taking concurrent temozolomide (1:1, 41%; 4:1, 31%), four (4) participants were prescribed both Lomustine and Bevacizumab (1:1, 0%; 4:1, 9.5%) and two (2) prescribed Bevacizumab only (1;1, 0%; 4:1 14.3%). Of the participants, 28 had been administered dexamethasone (1:1, 34%; 4:1 33.3%), 14% had in-operatable GBM’s (1:1, 14.6%; 4:1, 14.2%), 50.6% had one craniotomy (1:1, 53.6%; 4:1, 47.6%) and 34.3% had 2 or more craniotomies (1:1, 29%; 4:1, 38.5%). (see **Table 1**).

**Table 1:** Basic demographics of study participants.

Demographic and AnthropometricCharacteristic		Ratio				p-value
		1:1 (n=41)	4:1 (n=42)	Total (n=83)	
		***%***	***%***	***%***	
**Gender**					
	Male	43.9	57.1	50.6		0.228
	Female	56.1	42.9	49.4	
**Ethnicity**					
	Caucasian	90.2	95.2	92.8		0.380
	Others	9.8	4.8	7.2	
**ECOG Score**					
	0	70.7	47.6	59	
	1	17	17	17	
	2	4.5	14.2	9.6	
	3	7.3	21.4	14.5	
**Tumour Type**					
	GBM	88	95	90	
	AA3r	12	5	10	
		***Mean (SD)***	***Mean (SD)***	***Mean (SD)***	
**Age**		52.1 (12.7)	54.4 (12.6)	53.3 (12.6)		0.803
**Weight**		75.7 (19.4)	74.1 (22)	74.9 (20.6)		0.734
**BMI**		25.8 (5.5)	25.6 (5.8)	25.7 (5.6)		0.899
		***%***	***%***	***%***	
**Treatments**					
	Temozolomide	17 (41.5%)	13 (31%)	30 (36.1%)	
	Bevacizumab	0 (0%)	6 (14.3%)	6 (7.2%)	
	Lomustine	0 (0%)	4 (9.5%)	4 (4.8%)	
	Dexamethasone	14 (34%)	14 (33.3%)	28 (33.7%)	
	Craniotomies prior to trial 0	6 (14.6%)	6 (14.2%)	12 (14%)	
	1	22 (53.6%)	20 (47.6%)	42 (50.6%)	
	2	9 (22%)	12 (28.5%)	21 (25.3%)	
	3	2 (5%)	2 (5%)	4 (5%)	
	4	1 (2.4%)	2 (5%)	3 (4%)	

Of the 92 patients screened, 91.3% (n=84) had a diagnosed recurrent or inoperable GBM brain tumour and 8.7% (n=8) had diagnosed AA3 recurrent. The ECOG scores from the 88 participants enrolled included 61.4% (n=54) having a score of 0, 17% (n=15) had a score of 1, 10.2% (n=9) had a score of 2 and 13.4% (n=12) had a score of 3 due to all subjects being in a wheelchair. No participants had a score of 4 on the ECOG scale.

### Tolerability Results

The primary outcome of this trial was tolerability and both cannabis product ratios were found to be well tolerated. Three (3.4%) participants had their dose reduced due to side effects which included shaking and hallucinations at night with resolution of side effects following dose reduction [1:1 reduced from 3.8ml (THC 17.5mg:CBD 18.2mg) to 3ml (THC 13.8mg:CBD 14.4mg); 1:1 reduced from 2.1 (THC 9.7mg:CBD 10mg) to 1.5ml (THC 6.9mg:CBD 7.2mg); 4:1 reduced from 2.6ml (THC 39mg:CBD 9.9mg) to 1.5ml (THC 22.5mg:CBD 5.7mg) to 0.5ml (THC 7.5mg:CBD 1.9mg)].

The average dose tolerated in the 1:1 ratio was 2.25ml daily (THC 10.35mg:CBD 10.8mg), and for the 4:1 the average dose was 1.8ml daily (THC 27mg:CBD 6.8mg). Blood samples for each time point were collected and are currently being analysed for cannabinoids, cannabinoid metabolites and endocannabinoids to ensure compliance and evaluate contamination.

Expanding the analysis to the dose and amount of the tolerated cannabis compared to treatment outcome from the MRI results found that the participants with stable disease over the study period were equal numbers of females (n=9) and males (n=9) around 54 years old, and were primarily using the 1:1 ratio (n=18) with an average dose of 2.2ml daily (THC 12.4mg, CBD 12.9mg). For participants who had a reduction in disease, there were equal numbers for both the 1:1 (n=4) and 4:1 ratio (n=4). The 1:1 ratio had on average a dose of 2.8ml daily (THC 13.9, CBD 9.8) mostly male (n=3+ and around 59 years old. The 4:1 had on average a dose of 1.9ml daily (THC 22.2mg, CBD 8,.5mg), all female around 48 years of age. In the participant who had progressive disease, the majority were in the 4:1 ratio arm (n=14), had an average dose of 1.8ml daily (THC 27.5mg, CBD 6.9mg), and were mostly female (n=8) around 56 years old. (see **Table 2**)

**Table 2:** Group and Dose response to disease outcome over 12 weeks.

Disease Status	Group	Number (n=62) %	Age	Sex	Daily Dose	THC	CBD
			Years ± SD		(ml ± SD)	Mg ± SD	Mg ± SD
Stable disease	1:1	18 (29%)	53.9 ± 11.5	F=9	2.2 ± 0.7	12.4 ± 3.9	12.9 ± 4.1
			(min 33, max 74)	M=9			
	4:1	13 (21%)	51.6 ± 14	F=9	2 ± 0.5	28.7 ± 9.7	7.9 ± 2.3
			(min 21, max 68)	M=4			
Reduction in Disease	1:1	4 (6.4%)	59 ± 16	F=1	2.8 ± 0.9	13.9 ± 5.5	9.8 ± 5.9
			(min 35, max 69)	M=3			
	4:1	4 (6.4%)	48 ± 21.5	F=4	1.9 ± 0.3	22.2 ± 10.3	8.5 ± 2.8
			(min 22, max 73)	M=0			
Progressive disease	1:1	9 (14.5%)	53.9 ± 10.4	F=4	2.4 ± 0.9	17 ± 10.7	13 ± 3.7
			(min 34, max 66)	M=5			
	4:1	14 (22.5%)	56.3 ± 11.6	F=8	1.8 ± 0.9	27.5 ± 13.2	6.9 ± 3.3
			(min 26, max 74)	M=6			

The results of the paired t-test assessing difference in the FACT-Br between groups from baseline to week 12 found that both physical (p=0.025) and functional (p=0.014) domains were statistically significant in favour of the 1:1 ratio over the 4:1 ratio. Sleep (p=0.009) improved in both groups. (see **Table 3**). A longitudinal model generalized estimating equation (GEE) was used to assess all patients over each time point and found both the physical wellbeing (p=0.045) and sleep (p=0.012) were statistically significant (see **Table 4**).

**Table 3:** Paired T-test from Baseline to Week 12 Comparing 1:1 to 4:1 (FACT-BR).

	Mean Difference	95% Confidence Interval		p-value
	(1:1 group - 4:1 group)			
**FACT-BR**				
Total Fact Br	92.29	90.18	94.4	0.904
Total Physical	7.21	6.49	7.92	0.025
Total Social	21.4	20.76	22.19	0.653
Total Emotional	9.17	8.57	9.78	0.377
Total Functional	15.9	15	16.9	0.014
Total Other	38.5	37.32	39.74	0.229
**Quality of Life (QoL)**				
Pain	0.69	0.55	0.83	0.143
Sleep	2.59	2.4	2.77	0.009
Nausea	0.17	0.09	0.024	0.437
Anxiety	1.13	0.98	1.29	0.551
Seizures	0.51	0.37	0.67	0.336
Enjoyment of life	2.67	2.51	2.82	0.473
Physical lack of Energy	1.85	1.67	2.03	0.015
Content with QoL	2.18	2	2.36	0.006

**Table 4:** Mean scores (based on GEE analyses) of the outcome measures for the study participants in the two treatment groups over time.

Outcome	Group	Baseline	Week 4	Week 8	Week 12	p-value
		*Mean (SD)*	*Mean (SD)*	*Mean (SD)*	*Mean (SD)*	
**Fact-Br Total**	1:1	92.8 (19.1)	90.6 (15.9)	90.9 (15.7)	91.2 (12.6)	
	4:1	93.6 (13.3)	91.3 (14.2)	85.7 (15)	87.9 (17.1)	0.999
	Total	93.2 (16.3)	90.9 (14.9)	88.3 (15.5)	89.5 (15)	
**Physical**	1:1	6.4 (5.2)	5 (4.9)	6.4 (6.2)	6.4 (6.2)	
	4:1	8.5 (5.5)	8.4 (7.2)	7 (5.1)	6.5 (4)	0.045
	Total	7.4 (5.5)	6.7 (6.4)	6.7 (5.6)	6.5 (5.2)	
**Social**	1:1	21.3 (6.2)	22.5 (3.7)	22.3 (4.3)	22.5 (3.7)	
	4:1	21.6 (5.1)	20.3 (4.8)	20 (4.8)	20.4 (5.3)	0.420
	Total	21.5 (5.6)	21.4 (4.5)	21.1 (4.6)	21.4 (4.7)	
**Emotional**	1:1	9.8 (5)	8.05 (4.4)	7.8 (5)	8.4 (6.7)	
	4:1	9.3 (4.5)	8.2 (4.6)	7.1 (3.7)	7.7 (3.8)	0.713
	Total	9.6 (4.7)	8.1 (4.5)	7.5 (4.4)	8 (4)	
**Functional**	1:1	16.9 (7.5)	17.5 (6.3)	17.1 (5.7)	17.8 (6.8)	
	4:1	14.5 (6.9)	14.4 (5.9)	14.6 (7.1)	15.7 (6.3)	0.061
	Total	15.7 (7.3)	15.9 (6.3)	15.8 (6.7)	16.7 (6.6)	
**Brain**	1:1	38.3 (9.6)	37.5 (13.8)	37.3 (9.5)	36.1 (8.8)	
	4:1	39.8 (7.8)	39.3 (8.6)	36.5 (7.9)	37.5 (10.7)	0.333
	Total	39.1 (8.7)	38.4 (11.4)	36.9 (8.7)	36.8 (9.7)	
**Pain**	1:1	0.58 (0.92)	0.35 (07)	0.6 (0.1)	0.6 (.87)	
	4:1	0.76 (1.1)	0.77 (0.9)	0.76 (1.1)	0.89 (1.2)	0.234
	Total	0.67(1)	0.57 (0.86)	0.68 (1.1)	0.75 (1.1)	
**Sleep**	1:1	2.6 (1.4)	3.4 (0.9)	3.6 (0.7)	3.4 (0.9)	
	4:1	2.1 (1.4)	2.7 (1.2)	3.1 (0.87)	3.2 (0.8)	0.012
	Total	2.3 (1.4)	3 (1.1)	3.3 (0.82)	3.3 (0.87)	
**Nausea**	1:1	0.43 (0.74)	0.35 90.74)	0.56 (0.1)	0.78 (1.1)	
	4:1	0.54 (0.94)	0.56 (0.1)	0.74 (0.1)	0.55 (0.73)	0.068
	Total	0.5 (0.84)	0.46 (0.85)	0.67 (0.1)	0.67 (0.95)	
**Anxiety**	1:1	1.2 (1.2)	0.82 (0.91)	0.94 (1.2)	0.78 (0.9)	
	4:1	1.3 (1.2)	0.8 (0.9)	0.8 (0.93)	0.75 (0.9	0.816
	Total	1.3 (1.3)	0.81 (0.9)	0.86 (1.1)	0.77 (0.98)	
**Seizures**	1:1	0.51 (1.1)	0.41 (0.9)	0.5 (1.1)	0.25 (0.8)	
	4:1	0.67 (1.2)	0.42 (1)	0.2 (0.71)	0.34 (0.85)	0.701
	Total	0.6 (1.1)	0.42 (0.96)	0.34 (0.92)	0.29 (0.84)	
**Enjoyment of life**	1:1	2.7 (1.1)	2.7 (1.2)	2.9 (1)	2.7 (1.1)	
	4:1	2.6 (1.3)	2.5 (1)	2.3 (1.1)	2.6 (0.98)	0.222
	Total	2.6 (1.2)	2.6 (1.1)	2.6 (1.1)	2.7 (1)	
**Content with QoL**	1:1	2.4 (1.4)	2.5 (1.2)	2.4 (1.1)	2.5 (1.3)	
	4:1	1.9 (1.4)	1.8 (1.3)	2.1 (1.4)	1.9 (1.3)	0.027
	Total	2.2 (1.4)	2.1 (1.3)	2.2 (1.3)	2.2 (1.3)	

**Figure 2:**
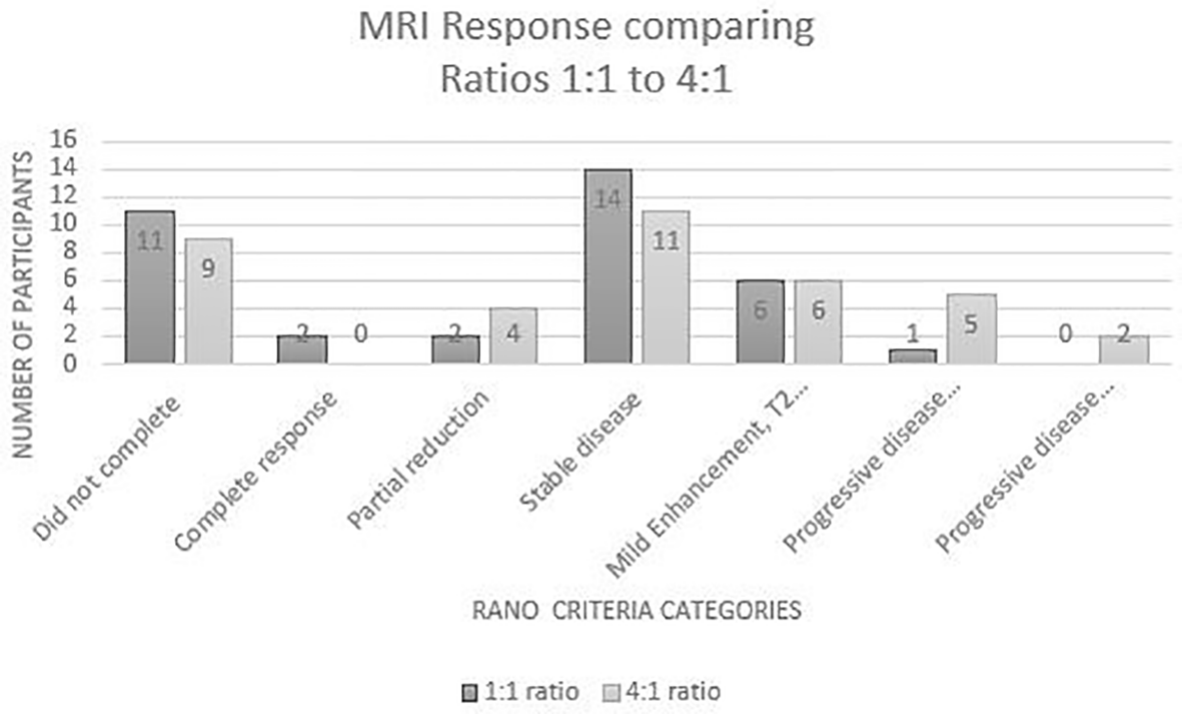
MRI responses from Baseline to Week 12.

For the secondary outcome, disease status was assessed through comparison of MRI results. In accordance with the RANO criteria, the results of the participants who had an MRI at baseline and week 12 (n=53) identified that 11% had a reduction in disease, 34% had stable disease, 16% had a T2 flair and slight enhancement and 10% had progressive disease. (see **Figure 2**) A chi square analysis was conducted between groups for each disease status which found no statistical significance. Three of the disease states were the same value or too small to evaluate a difference. This indicates there is no difference between the 1:1 or the 4:1 for MRI tumour burden/control or reduction in relation cannabis ratios for disease status. (see **Table 5**).

**Table 5:** Comparison between the two groups from MRI results with Chi Square analysis.

RANO Criteria (Baseline to 12 weeks)	Total	1:1	4:1	Chi^2^
	(n=53)	(n=25)	(n=28)	(p value)
Reduction in tumour size	8 (10.9%)	4 (16%)	4 (14.3%)	1.0
Stable disease	25 (34.1%)	14 (56%)	11 (39.3%)	0.751
Slight enhancement (T2 flair)	12 (16%)	6 (24%)	6 (21.4%)	1.0
≤25% growth – progressive disease	6 (8%)	1 (4%)	5 (17.8%)	0.137
≥25% growth – progressive disease	2 (2%)	0 (0%)	2 (7.1%)	1.0

### Retrospective Control Group

The retrospective case data (n=61) included a greater proportion of dexamethasone administration (cannabis 33.7% *vs* 91% in retrospective data) and craniotomies in the control group compared with the study sample. The results of the cannabis group *versus* the retrospective data found 11% reduction in tumour size in cannabis arm *versus* 0% in retrospective data, 34% stable disease *versus* 45% stable disease, and 27.5% *versus* 53% for progressive disease.

### Safety and Adverse Events

No serious adverse events were found to be attributable to the intervention during the trial. The main side effects noted were dry mouth (13%), tiredness at night (11%), dizziness mainly at night (10%), and drowsiness (7%). There were no reports of psychosis although at week 8, four participants (6%) reported mild hallucinations, some paranoia or euphoria at night. The number of total side effects identified decreased during the trial from 57% to 41% by week 12. (see **Table 6**).

**Table 6:** Side effects from the cannabis interventions as reported by the study participants.

Side Effects	Cannabis Ratio Group	Week 4 (n=88)	Week 8 (n=71)	Week 12 (n=61)
		1:1 = 45	1:1 = 34	1:1 = 29
		4:1 = 43	4:1 = 37	4:1 = 32
Total Side Effects	1:1	22 (49%)	15 (44%)	12 (41%)
	4:1	28 (65%)	23 (62%)	13 (40%)
	Total	50 (57%)	38 (53.5%)	25 (41%)
Dry Mouth	1:1	6 (13%)	5 (15%)	4 (14%)
	4:1	7 (16%)	6 (16%)	4 (12.5%)
	Total	13 (15%)	11 (15.5%)	8 (13%)
Tiredness	1:1	4 (9%)	4 (12%)	0 (0%)
	4:1	8 (18.6%)	6 (16%)	5 (15.6%)
	Total	11 (12.5%)	10 (14%)	5 (8%)
Dizziness	1:1	1 (2%)	3 (9%)	2 (7%)
	4:1	6 (14%)	4 (11%)	4 (12.5%)
	Total	7 (8%)	7 (10%)	6 (10%)
Drowsiness	1:1	1 (2%)	1 (3%)	0 (0%)
	4:1	6 (14%)	4 (11%)	1 (3%)
	Total	7 (8%)	5 (7%)	1 (1.6%)
Impaired motor coordination	1:1	5 (11.6%)	2 (6%)	1 (3.5%)
	4:1	2 (4%)	1 (3%)	0 (0%)
	Total	7 (8%)	3 (4%)	1 (1.6%)
Seizures	1:1	2 (4%)	1 (3%)	1 (3.5%)
	4:1	3 (7%)	3 (8%)	1 (3%)
	Total	5 (6%)	4 (6%)	2 (3%)
Headaches	1:1	0 (0%)	0 (0%)	2 (7%)
	4:1	1 (2%)	2 (5.4%)	2 (6%)
	Total	1 (1%)	2 (3%)	4 (6.5%)
Nausea	1:1	2 (4%)	3 (9%)	2 (7%)
	4:1	1 (2%)	5 (13.5%)	1 (3%)
	Total	3 (3.5%)	8 (11%)	3 (5%)
Increased appetite	1:1	1 (2%)	0 (0%)	0 (0%)
	4:1	2 (4%)	1 (3%)	3 (9%)
	Total	3 (3.5%)	1 (1.5%)	3 (5%)
Increased urination at night	1:1	2 (4%)	1 (3%)	0 (0%)
	4:1	1 (2%)	0 (0%)	0 (0%)
	Total	3 (3.5%)	1 (1.5%)	0 (0%)
Constipation	1:1	0 (0%)	0 (0%)	0 (0%)
	4:1	2 (4%)	0 (0%)	1 (3%)
	Total	2 (2%)	0 (0%)	1 (1.6%)
Increased urination at night	1:1	2 (4%)	1 (3%)	0 (0%)
	4:1	1 (2%)	0 (0%)	0 (0%)
	Total	3 (3.5%)	1 (1.5%)	0 (0%)
Hallucinations/ Paranoia/euphoria	1:1	0 (0%)	2 (6%)	1 (3.5%)
	4:1	0 (0%)	2 (5.4%)	1 (3%)
	Total	0 (0%)	4 (6%)	2 (3%)
Psychosis	1:1	0 (0%)	0 (0%)	0 (0%)
	4:1	0 (0%)	0 (0%)	0 (0%)
	Total	0 (0%)	0 (0%)	0 (0%)

The main findings from the NCI-CTC for all participants who completed the 12 weeks were bloating (p=0.034), shortness of breath (p=0.012), dry skin (p=0.034), visual floaters (p=0.037), concentration (p=0.033), muscle pain (p=0.019), and insomnia (p=0.011) (see **Supplemental Information**).

No statistically significant difference in blood pathology tests was identified between baseline to week 12 over time (see **Supplemental Information**). Serum liver enzymes were regularly checked *via* liver function tests (LFT) for all participants who were on specific treatments for high-grade gliomas. When associations between liver enzymes and the use of anticancer therapy were tested, a statistically significant association between raised GGT and temozolomide (n=30; p=0.028) was identified. Also, small number of participants (n=6) had been prescribed bevacizumab and a statistically significant association with elevated ALP and GGT was found in this group. Similarly, the analysis identified a statistically significant association between lomustine patients on Lomusine (n=4) and raised ALP (p=0.001).

## Discussion

In this study, we performed a blinded, phase 2 trial administering MC to patients with high grade gliomas. Patients were randomized to one of two formulations with differing THC : CBD ratios. Currently, there are multiple studies explore the anti-inflammatory and anti-cancer potential of MC for patients with high grade gliomas. However, such research also relies on reliable information regarding tolerability and dosing before efficacy trials can be conducted. Currently this evidence is lacking in the literature.

The mechanism of action of CBD and THC is important for deciding which ratios are useful for particular diseases and symptoms (67). CBD is well known as the non-intoxicating cannabinoid compared to THC. However, it is the THC that binds to CB1 receptors which are mainly located in the central nervous system, predominately the brain, in addition to other organs and tissues (68). *In vitro* and animal studies have identified that cannabis has anti-tumoral activity and xenografts on rats and mice have shown that it can reduce high grade glioma growth (69, 70). Pellati et al. noted that both CBD and THC demonstrated anti-inflammatory activity by downregulation of the proto-oncogene c-fos and clycooxygenase-2 (COX-2) and potential anti-tumoral activity (71). Other *in vitro* models of neuroinflammation on rat microglia, found that CBD suppresses tumour necrosis factor alpha (TNF-α), interleukin (IL)-1β and IL-6, reduces nuclear factor kappa beta (NF-*κ*B) phosphorylation and activates COX and inducible nitric oxide synthase (iNOS) (72). CBD also causes a downregulation of Akt and ERK pathways in human glioma cells (73).

We propose this study provides robust evidence that medicinal cannabis administered to this patient population is safe, well tolerated, and can provide symptomatic relief to these patients, improving their QoL. While the overall blood brain barrier (BBB) penetration and general safety of cannabis has been demonstrated in healthy populations (74), this study is one of the first to evaluate the safety and tolerability of MC in this vulnerable cancer population, a patient group with BBB disruption, impaired brain function, and numerous medications including chemotherapy, corticosteroids, and antiepileptics.

Our study data suggests that cannabis, especially a 1:1 CBD/THC mixture can be helpful for many of the symptoms impacting QoL in this patient population, especially sleep disturbance. As such, MC may be a valuable potential therapy for maintaining the best QoL and daily function for this poor prognosis population, whist also assisting patients during anticancer and potential life extending therapies. High grade brain tumours, and their treatments, are well known to cause a constellation of symptoms, especially nausea from radiotherapy and chemotherapy, cerebral oedema or inflammation (75), increased intracranial pressure and sleep disturbance (or insomnia). This in part may be due to medication related side effects, notably corticosteroids in addition to radiation and chemotherapy (76). QoL concerns in patients with malignant high grade gliomas are relevant to more than just palliative care physicians, but have real oncologic and survival consequences (77).

Given the inevitability of tumour recurrence in these patients (78), patients with poorer QoL, higher symptom burdens and poorer functional status are less likely to elect for additional therapy, which often can extend their life (76). In this context, our study provides an important platform for further trials to explore the 1:1 mixture in larger cohorts.

## Future Directions

This trial allowed a mixture of patients with different tumour grades, different disease stages (advanced, recurrent tumours and inoperable tumours), and did not sub-stratify patients by molecular markers, such as IDH-1 mutation. This style of recruitment was conducted to gather data for further trials to ascertain which cohort will be best to focus on for efficacy trials. The tolerability and improved symptomatic benefit for these patients encourages the incorporation of MC in future well designed, randomized-controlled trials that include cannabis-free controls, for the treatment of patients with high grade gliomas.

It is also important to note, that while generally we did not identify any adverse events related to drug interactions between MC and drugs commonly administered to high-grade glioma patients, notably chemotherapy and antiepileptics, we cannot exhaustively conclude that adding cannabis to the regimen of glioma patients will never lead to adverse drug interactions. Notably, the number of coadministration of MC with bevacizumab and lomustine was too low to meaningfully conclude this combination is safe in these patients. However, despite this limitation we feel this study supports the idea that it is reasonably safe to explore this further in this patient cohort.

## Limitations

There were several limitations. Firstly, there was no placebo group which is considered gold standard in randomised-clinical trials and the historical retrospective data which was used as a comparison was compromised as the client population for the clinician providing the retrospective sample were commonly in advanced stages of cancer and hence many were excluded from the analysis. The difference in treatments used in the retrospective cases and the study population also limits the comparison between these groups. The length of time of the intervention (12 weeks) also limits the conclusions which can be drawn from this study and future research should consider longer time frames. However, longitudinal studies are continuing for this trial with up to two years follow up of the cohort. This trial was also very pragmatic, rather than controlled, to give as broad a clinically useful picture as possible. As such, this limits the strength of the data due the heterogeneity of the cohort and the variations in concurrent treatments. In saying that, real life pragmatic trials are necessary for translation and implementation and can give clinicians a much clearer patient picture for everyday care. In addition, a strong intervention affect can be expected for the MC group as there was no placebo. It would be advised that a placebo-controlled trial be conducted in the future to ascertain accurate data.

## Conclusion

Addressing the symptom clusters, improving symptoms and in turn QoL is the first and crucial step in improving health outcomes for this population of cancer patients. This remains a vulnerable patient group with rapid deterioration, poor QoL and poor prognosis. Despite increasing interest in the efficacy in disease control of MC in this population, associated research examining the tolerability and safety of MC products is scarce. From this study we have shown that a single nightly dose of THC-containing cannabis was well tolerated in patients in both groups with high-grade gliomas and significantly improved sleep, functional wellbeing and contentment with QoL in a sample of patients compared to baseline. From this trial, the 1:1 ratio has been identified as the preferred combination the moving forward to further trials. This study significantly informs MC product choice for ongoing studies into cannabis being a potential adjunct treatment option for this patient population.

## Study Sponsors

Endeavour College of Natural Health, 269 Wickham Street, Fortitude Valley, Qld 4006 NCNM, Southern Cross University, Military Road, Lismore NSW. NB: The funding body was not involved in the study design, collection, analysis, interpretation of data, the writing of this article or the decision to submit it for publication.

## Data Availability Statement

The raw data supporting the conclusions of this article can be made available upon request by the authors, without undue reservation.

## Ethics Statement

The studies involving human participants were reviewed and approved by South Eastern Sydney Local Health District HREC. The patients/participants provided their written informed consent to participate in this study.

## Author Contributions

JaS was the lead for the manuscript with all authors contributed to the paper in addition to editing final versions. All authors contributed to the article and approved the submitted version.

## Funding

This study was supported financially by FIT-Bioceuticals Pty Ltd, Sydney, Australia. The funder bodies were not involved in the study design, collection, analysis, interpretation of data, the writing of this article or the decision to submit it for publication.

## Conflict of Interest

The authors declare that the research was conducted in the absence of any commercial or financial relationships that could be construed as a potential conflict of interest.
