# Supplementary material for: A Phase 2 Randomised Clinical Trial Assessing the Tolerability of Two Different Ratios of Medicinal Cannabis in Patients With High Grade Gliomas
Source: Front Oncol. 2021 May 21;11:649555. doi: 10.3389/fonc.2021.649555 (PMC8176855; doi:10.3389/fonc.2021.649555)
Supplement: Supplementary file 1 [file DataSheet_1.pdf]

Supplementary Material

A PHASE 2 RANDOMISED, DOUBLE BLIND CLINICAL TRIAL ASSESSING THE TOLERABILITY OF TWO DIFFERENT RATIOS OF MEDICINAL CANNABIS IN PATIENTS WITH GLIOBLASTOMA MULTIFORME (GBM).

Supplemental information

1. Retrospective data comparison
2. Scores of blood pathology
3. Score of treatment related toxicity

RANO Criteria (Baseline to 12 weeks)	MC Group	Retro Group
Reduction in tumour size	8 (10.9%)	0 (0%)
Stable disease	25 (34.1%)	29 (45%)
Progressive disease	20 (27.4%)	34 53%)

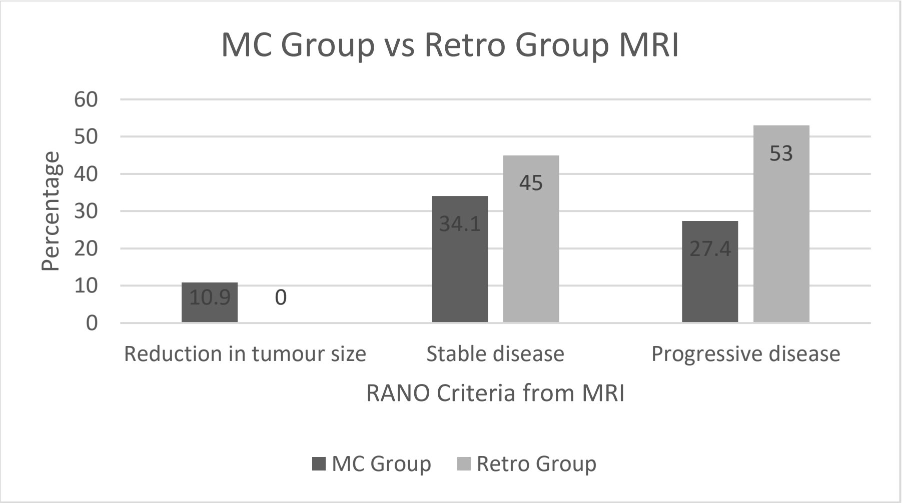

Supplementary Figure 1: Comparison of MRI results after 12 weeks

Supplementary Table 1: Scores of blood pathology markers (GEE, paired t test for Mean and SD)

No correlation between those who were taking phenytoin and carbinamine with blood levels.

Outcome		Baseline	Week 4	Week 8	Week 12	p- value
		Mean (SD)	*Mean (SD)*	*Mean (SD)*	*Mean (SD)*	
**Sodium**	1:1	142.3 (2.7)	142.0 (2.6)	141.6 (2.8)	142.1 (2.3)	
	4:1	141.4 (2.6)	141.5 (2.0)	141.0 (1.9)	141.7 (1.4)	0.235
(135-145)	Total	141.8 (2.8)	141.7 (2.3)	141.3 (2.4)	141.9 (1.8)	
**Potassium**	1:1	5.25 (6.7)	4.12 (0.3)	4.06 (0.4)	1.14 (0.4)	
	4:1	4.23 (0.2)	4.10 (0.3)	4.06 (0.4)	3.97 (0.2)	0.306
(3.5-5.2)	Total	4.75 (4.8)	4.11 (0.3)	4.06 (0.4)	4.05 (0.3)	
**Chloride**	1:1	102.4 (3.2)	101.8 (2.7)	101.6 (2.8)	102.8 (2.1)	
	4:1	101.5 (2.6)	101.7 (2.6)	101.0 (2.4)	101.6 (2.5)	0.271
(95-110)	Total	102.0 (2.9)	101.8 (2.6)	101.3 (2.6)	102.2 (2.4)	
**HC03**	1:1	26.27 (2.3)	25.42 (2.2)	25.6 (1.8)	24.5 (2.0)	
	4:1	25.33 (2.2)	24.44 (2.2)	24.0 (4.4)	26.0 (2.0)	0.067
(22-32)	Total	25.81 (2.3)	24.90 (2.2)	24.8 (3.5)	25.3 (2.1)	
**Urea**	1:1	5.3 (1.4)	5.8 (1.5)	5.4 (1.8)	5.5 (1.5)	
	4:1	5.5 (2.0)	5.6 (1.8)	5.4 (1.8)	5.1 (1.9)	0.358
(4.0-9.0)	Total	5.4 (1.7)	5.7 (1.7)	5.4 (1.7)	5.3 (1.7)	
**Creatinine**	1:1	73 (22.6)	69.4 (19.8)	72.4 (16.4)	71.5 (18.1)	
	4:1	72.5 (18.2)	70.0 (13.0)	71.7 (16.9)	70.2 (11.5)	0.222
(60-110)	Total	72.7 (20.4)	69.7 (16.4)	72.0 (16.5)	70.8 (14.8)	
**EGFR**	1:1	86.6 (9.2)	86.3 (7.7)	86.3 (6.5)	86.2 (6.8)	
	4:1	84.3 (9.9)	86.5 (5.6)	84.4 (10.1)	85.2 (7.2)	0.387
(>90)	Total	85.5 (9.6)	86.4 (6.7)	85.3 (8.6)	85.6 (7.0)	
**Glucose**	1:1	5.5 (1.2)	5.2 (1.5)	5.0 (1.3)	5.4 (1.2)	
	4:1	5.6 (1.6)	5.5 (1.3)	5.6 (2.1)	4.9 (0.8)	0.323
(3.0-5.5)	Total	5.5 (1.4)	5.3 (1.4)	5.3 (1.8)	5.1 (1.0)	
**Bilirubin**	1:1	8.2 (5.7)	7.6 (3.3)	9.1 (6.6)	7.4 (3.5)	
	4:1	6.7 (4.3)	7.0 (4.3)	7.4 (5.7)	7.1 (4.4)	0.149
(0-20)	Total	7.5 (5.1)	7.2 (3.8)	8.2 (6.7)	7.2 (4.0)	
**ALP**	1:1	71.3 (26.5)	65.0 (19.6)	68.5 (18)	66.1 (15.2)	
	4:1	72.6 (27.2)	71.4 (35.2)	67.3 (24)	71.4 (25.3)	0.661
(30-110)	Total	71.9 (26.7)	68.5 (29.1)	67.8 (21)	68.8 (21.0)	
**GGT**	1:1	40.2 (44.3)	33.0 (32.6)	38.0 (49)	28.9 (40.4)	

	4:1	48.5 (51.8)	42.4 (69.8)	43.6 (65)	30.7 (31.5)	0.462
(5-50)	Total	44.3 (48.0)	38.1 (55.8)	41.0 (58)	29.8 (35.6)	
**AST**	1:1	22.8 (14.3)	20.2 (7.2)	22.9 (13)	19.3 (7.5)	
	4:1	22.1 (8.1)	20.9 (9.5)	20.1 (6.9)	20.6 (7.4)	0.545
(<36)	Total	22.5 (11.6)	20.6 (8.4)	21.4 (10)	20.0 (7.4)	
**ALT**	1:1	32.4 (31.5)	29.4 (24.1)	30.8 (28)	24.0 (21.7)	
	4:1	29.6 (18.0)	29.1 (23.8)	26.8 (19)	25.7 (14.4)	0.570
(<51)	Total	31.0 (25.6)	29.2 (23.7)	28.6 (24)	24.9 (18.0)	
**Protein**	1:1	67.7 (5.1)	66.8 (4.2)	66.5 (4.0)	67.0 (3.7)	
	4:1	68.8 (5.3)	67.6 (6.0)	68.3 (5.1)	68.1 (5.1)	0.250
(60-80)	Total	68.2 (5.2)	67.3 (5.2)	67.5 (4.7)	67.6 (4.5)	
**Albumin**	1:1	37.8 (7.1)	37.5 (5.6)	38.1 (3.6)	39.5 (2.0)	
	4:1	38.8 (3.6)	37.8 (3.2)	39.5 (3.3)	38.6 (3.6)	0.379
(33-48)	Total	38.3 (5.6)	37.7 (4.4)	38.9 (3.5)	39.1 (3.0)	
**CRP**	1:1	2.7 (3.8)	2.1 (2.4)	3.7 (7.1)	3.2 (5.8)	
	4:1	2.8 (4.2)	4.6 (7.3)	3.5 (6.4)	3.4 (4.6)	0.213
(<3)	Total	2.8 (4.0)	3.5 (5.7)	3.6 (6.7)	3.3 (5.1)	
**Hb**	1:1	138.4 (15)	136.4 (18)	136.8 (19)	134.1 (14.6)	
	4:1	133.2 (13)	130.1 (14)	129.8 (12)	127.9 (14.8)	0.063
(130-180)	Total	135.7 (14)	133.0 (16)	133.0 (15)	130.9 (14.9)	
**WBC**	1:1	6.3 (3.1)	6.6 (3)	6.1 (2.9)	5.3 (1.9)	
	4:1	5.5 (3.0)	5.5 (2)	5.2 (2.0)	5.0 (2.0)	0.189
(3.50-11.00)	Total	5.9 (3.1)	6.0 (2)	5.6 (2.4)	5.1 (2.0)	
**PLT**	1:1	197.9 (64)	217.5 (71)	198.5 (65)	202.1 (77.5)	
	4:1	190.5 (93)	195.1 (91)	189.7 (69)	179.0 (76.3)	0.423
(150-450)	Total	194.51(80)	205.5 (82)	193.8 (67)	190.3 (76.9)	
**RBC**	1:1	4.4 (0.4)	4.2 (0.8)	4.4 (0.6)	4.2 (0.5)	
	4:1	4.2 (0.5)	4.2 (0.6)	4.1 (0.5)	4.1 (0.6)	0.169
(4.50-6.50)	Total	4.3 (0.5)	4.2 (0.7)	4.3 (0.6)	4.1 (0.5)	
**HCT**	1:1	0.40 (0)	0.39 (0)	0.40 (0)	0.39 (0)	
	4:1	0.39 (0)	0.38 (0)	0.38 (0)	0.38 (0)	0.066
(0.40-0.54)	Total	0.39 (0)	0.38 (0)	0.39 (0)	0.39 (0)	
**MCV**	1:1	91.9 (4.3)	91.8 (4.8)	91.5 (4.8)	93.5 (4.5)	
	4:1	88.2 (17)	92.6 (5.8)	93.4 (6.6)	94.7 (7.0)	0.875
(80-100)	Total	90.0 (12)	92.2 (5.3)	92.5 (5.8)	94.1 (5.9)	
**MCH**	1:1	31.2 (1.7)	31.3 (1.9)	30.9 (2.2)	31.3 (1.7)	
	4:1	31.2 (2.3)	30.4 (4.3)	31.1 (2.8)	31.4 (2.6)	0.277

(26.5-33.0)	Total	31.2 (2.0)	30.8 (3.4)	31.0 (2.5)	31.4 (2.2)	
**MCHC**	1:1	339.9 (12)	340.6 (13)	334.8 (23)	335.3 (11.6)	
	4:1	338.8 (9)	337.0 (12)	332.9 (12)	331.8 (9.6)	0.644
(310-360)	Total	339.8 (10)	338.7 (13)	333.8 (18)	333.5 (10.6)	
**RDW_CV**	1:1	13.4 (0.8)	13.9 (1.2)	13.9 (1.3)	13.6 (1.1)	
	4:1	14.0 (1.2)	14.2 (1.6)	13.8 (1.3)	13.6 (1.2)	0.162
(12.0-14.5)	Total	13.8 (1.1)	14.0 (1.4)	13.8 (1.3)	13.6 (1.1)	
**RDWSD**	1:1	45.06 (2.8)	46.32 (4.3)	45.9 (4.3)	46.4 (4.7)	
	4:1	47.10 (5.5)	48.01 (6.6)	47.1 (6.0)	46.7 (5.0)	0.059
(38.0-48.0)	Total	46.11 (4.5)	47.22 (5.7)	46.5 (5.3)	46.6 (4.8)	
**MPV**	1:1	10.56 (1)	10.56 (1)	10.5 (1.0)	10.7 (1.0)	
	4:1	10.30 (1)	10.30 (1)	10.4 (0.8)	10.6 (0.8)	0.162
(7.4-11.7)	Total	10.43 (1)	10.43 (1)	10.4 (0.9)	10.6 (0.9)	
**Neutrophil**	1:1	6.37 (12)	6.67 (12.6)	4.25 (2.6)	3.68 (1.7)	
	4:1	3.94 (2.7)	3.94 (2.7)	3.74 (1.7)	3.36 (1.6)	0.259
(1.7-7.0)	Total	5.27 (9.0)	5.27 (9.0)	3.98 (2.2)	3.52 (1.6)	
**Lymphocytes**	1:1	0.35 (1.4)	1.35 (1.4)	1.28 (0.7)	1.10 (0.5)	
	4:1	1.10 (0.5)	1.10 (0.5)	1.08 (0.5)	1.20 (0.5)	0.153
(1.5-4.0)	Total	1.22 (1.0)	1.22 (1.0)	1.17 (0.6)	1.15 (0.5)	
**Monocyte**	1:1	0.47 (0.7)	0.47 (0.7)	0.39 (0.1.)	0.36 (0.1)	
	4:1	0.37 (0.2)	0.37 (0.2)	0.32 (0.1)	0.34 (0.1)	0.165
(0.1-0.8)	Total	0.42 (0.5)	0.42 (0.5)	0.36 (0.1)	0.35 (0.1)	
**Eosinophil**	1:1	0.25 (0.7)	0.25 (0.7)	0.16 (0.2)	0.16 (0.1)	
	4:1	0.09 (0.1)	0.09 (0.1)	0.10 (0.1)	0.10 (0.0)	0.153
(0.04-0.44)	Total	0.17 (0.5)	0.17 (0.5)	0.13 (0.1)	0.13 (0.1)	
**Basophil**	1:1	0.02 (0.0)	0.02 (0.0)	0.01 (0.0)	0.02 (0.0)	
	4:1	0.01 (0.0)	0.01 (0.0)	0.02 (0.0)	0.02 (0.0)	0.907
(0.0-0.2)	Total	0.01 (0.0)	0.01 (0.0)	0.02 (0.0)	0.02 (0.0)	

Outcome		Baseline	Week 4	Week 8	Week 12
		Mean (SD)	*Mean (SD)*	*Mean (SD)*	*Mean (SD)*
**Phenytoin**	1:1	8.9 (11.45)	4.1	10	N/A
	4:1	0.8	1.1	1.9	N/A
	Total	6.2	3.5	5.9	
**Carbamazepine**	1:1	2 (0)	1.6	N/A	N/A
	4:1	2 (0)	2	N/A	N/A
	Total	2 (0)	1.7		

Supplementary Table 2: Treatment related toxicity – NCI CTC
Comparison of toxicity results between participants: (GEE, paired t test for Mean and SD)

Outcome		Baseline	Week 4	Week 8	Week 12	p- value
		Mean (SD)	*Mean (SD)*	*Mean (SD)*	*Mean (SD)*	
**Dry Mouth**	1:1	0.63 (0.96)	0.76 (0.80)	0.73 (0.89)	0.67 (0.90)	
	4:1	0.70 (0.9)	1.07 (0.97)	0.88 (0.97)	0.78 (0.95)	0.213
	Total	0.67 (0.93)	0.92 (0.90)	0.81 (0.93)	0.73 (0.92)	
**Difficulty swallowing**	1:1	0.17 (0.49)	0.28 (0.60)	0.23 (0.55)	0.17 (0.39)	
	4:1	0.19 (0.39)	0.37 (0.66)	0.30 (0.57)	0.28 (0.59)	0.387
	Total	0.18 (0.44)	0.32 (0.63)	0.27 (0.56)	0.23 (0.50)	
**Mouth sores**	1:1	0.09 (0.37)	0.05 (0.22)	0.11 (0.32)	0.25 (0.58)	
	4:1	0.14 (0.47)	0.17 (0.44)	0.05 (0.33)	0.25 (0.70)	0.626
	Total	0.12 (0.42)	0.11 (0.35)	0.08 (0.32)	0.25 (0.63)	
**Mouth sores interference**	1:1	0.14 (0.57)	0.02 (0.16)	0.08 90.28)	0.14 (0.60)	
	4:1	0.07 (0.46)	0.00 (0.00)	0.08 (0.50)	0.18 (0.68)	0.662
	Total	0.10 (0.51)	0.01 (0.11)	0.08 (0.40)	0.16 (0.63)	
**Cheilosis**	1:1	0.04 (0.31)	0.05 (0.22)	0.14 (0.55)	0.07 (0.26)	
	4:1	0.11 (0.32)	0.10 (0.30)	0.08 (0.36)	0.03 (0.18)	0.842
	Total	0.08 (0.32)	0.07 (0.26)	0.11 (0.46)	0.05 (0.22)	
**Voice changes**	1:1	0.14 (0.35)	0.05 (0.22)	0.08 (0.28)	0.17 (0.39)	
	4:1	0.19 (0.39)	0.15 (0.36)	0.16 (0.37)	0.22 (0.42)	0.195
	Total	0.16 (0.37)	0.10 (0.30)	0.12 (0.33)	0.20 (0.40)	
**Hoarseness**	1:1	0.19 (0.45)	0.12 (0.40)	0.17 (0.38)	0.28 (0.53)	
	4:1	0.40 (0.79)	0.20 (0.40)	0.16 (0.50)	0.28 (0.71)	0.350
	Total	0.30 (0.65)	0.16 (0.40)	0.17 (0.44)	0.28 (0.62)	
**Taste changes**	1:1	0.21 (0.41)	0.28 (0.64)	0.32 (0.68)	0.17 (0.47)	
	4:1	0.38 (0.69)	0.32 (0.69)	0.55 (0.84)	0.50 (0.92)	0.152
	Total	0.30 (0.57)	0.30 (0.66)	0.44 (0.77)	0.33 (0.74)	
**Reded appetite**	1:1	0.58 (0.83)	0.54 (0.75)	0.73 (0.93)	0.42 (0.69)	
	4:1	0.59 90.85)	0.67 (0.97)	0.80 (1.23)	0.60 (0.78)	0.684
	Total	0.59 (0.84)	0.61 (0.86)	0.77 (1.09)	0.51 (0.73)	
**Appetite interference**	1:1	0.29 (0.64)	0.23 (0.54)	0.37 (0.83)	0.28 (0.65)	
	4:1	0.21 (0.47)	0.13 (0.52)	0.32 (0.58)	0.37 (0.74)	0.515
	Total	0.25 (0.55)	0.18 (0.53)	0.34 (0.71)	0.32 (0.69)	
**Nausea**	1:1	0.65 (0.79)	0.53 (0.82)	0.79 (1.12)	0.92 (1.29)	

	4:1	0.57 (0.88)	0.75 (0.98)	0.91 (0.96)	0.59 (0.93)	0.958	
	Total	0.61 (0.83)	0.64 (0.90)	0.85 (1.03)	0.75 (1.13)		
**Nausea severity**	1:1	0.48 (0.63)	0.47 (0.72)	0.52 (0.74)	0.57 (0.79)		
	4:1	0.45 (0.77)	0.62 (1.00)	0.83 (0.87)	0.60 (0.99)	0.448	
	Total	0.46 (0.70)	0.55 (0.87)	0.68 (0.82)	0.58 (0.88)		
**Vomiting**	1:1	0.19 (0.55)	0.23 (0.53)	0.14 (0.50)	0.25 (0.64)		
	4:1	0.19 (0.45)	0.12 (0.33)	0.19 (0.52)	0.17 (0.47)	0.551	
	Total	0.19 (0.50)	0.17 (0.44)	0.17 (0.50)	0.21 (0.56)		
**Vomiting severity**	1:1	0.17 (0.49)	0.23 (0.58)	0.08 (0.37)	0.28 (0.65)		
	4:1	0.16 (0.48)	0.15 (0.42)	0.20 (0.53)	0.11 (0.42)	0.606	
	Total	0.16 (0.48)	0.19 (0.51)	0.14 (0.46)	0.20 (0.55)		
**Heart burn**	1:1	0.43 (0.70)	0.38 (0.81)	0.29 (0.57)	0.32 (0.77)		
	4:1	0.35 (0.61)	0.52 (0.87)	0.55 (0.73)	0.35 (0.67)	0.412	
	Total	0.39 (0.66)	0.45 (0.84	0.42 (0.67)	0.33 (0.72)		
**Heart burn severity**	1:1	0.29 (0.51)	0.30 (0.73)	0.24 (0.50)	0.21 (0.56)		
	4:1	0.30 (0.60)	0.40 (0.70)	0.41 (0.60)	0.25 (0.51)	0.350	
	Total	0.30 (0.55)	0.35 (0.71)	0.33 (0.56)	0.23 (0.53)		
**Gas**	1:1	0.09 (0.30)	0.15 (0.36)	0.26 (0.51)	0.17 (0.39)		
	4:1	0.16 (0.37)	0.25 (0.43)	0.05 (0.23)	0.17 (0.39)	0.848	
	Total	0.13 (0.34)	0.20 (0.40)	0.15 (0.40)	0.17 (0.38)		
**Bloating**	1:1	0.56 (0.86)	0.36 (0.75)	0.73 (0.96)	0.53 (0.74)		
	4:1	0.78 (1.11)	0.80 (1.18)	0.66 (1.01)	0.35 (0.78)	0.209	
	Total	0.67 (1.00)	0.58 (1.01)	0.70 (0.98)	0.44 (0.76)		
**Bloating severity**	1:1	0.35 (0.57)	0.35 (0.70)	0.35 (0.59)	0.32 (0.47)		
	4:1	0.65 (0.85)	0.62 (0.89)	0.52 (0.73)	0.25 (0.64)	0.034	4:1
	Total	0.50 (0.74)	0.49 (0.81)	0.44 (0.67)	0.28 (0.56)		
**Hiccups**	1:1	0.32 (0.65)	0.42 (0.68)	0.47 (0.86)	0.50 (0.83)		
	4:1	0.38 (0.79)	0.57 (0.90)	0.55 (0.99)	0.28 (0.59)	0.743	
	Total	0.35 (0.72)	0.50 (0.80)	0.51 (0.92)	0.39 (0.73)		
**Hiccups severity**	1:1	0.25 (0.58)	0.26 (0.50)	0.35 (0.64)	0.42 (0.69)		
	4:1	0.21 (0.51)	0.35 (0.69)	0.38 (0.72)	0.21 (0.49)	0.930	
	Total	0.23 (0.55)	0.30 (0.60)	0.37 (0.68)	0.32 (0.60)		
**Constipation**	1:1	0.67 (0.82)	0.41 (0.71)	0.70 (1.08)	0.50 (0.69)		
	4:1	0.61 (0.93)	0.77 (0.91)	0.41 (0.69)	0.64 (0.86)	0.660	
	Total	0.64 (0.88)	0.59 (0.84)	0.55 (0.91)	0.57 (0.78)		

**Diarrhoea**	1:1	0.39 (0.73)	0.36 (0.71)	0.61 (0.77)	0.53 (0.69)		
	4:1	0.54 (0.73)	0.50 (0.84)	0.55 (0.96)	0.64 (1.06)	0.376	
	Total	0.46 (0.73)	0.43 (0.78)	0.58 (0.87)	0.58 (0.88)		
**Ab Pain Frequency**	1:1	0.53 (0.89)	0.41 (0.81)	0.47 (.066)	0.21 (0.49)		
	4:1	0.52 (0.83)	0.62 (0.83)	0.69 (0.95)	0.32 (0.66)	0.217	
	Total	0.53 (0.86)	0.51 (0.82)	0.58 (0.82)	0.26 (0.58)		
**Ab pain severity**	1:1	0.41 (0.66)	0.30 (0.56)	0.44 (0.22)	0.25 (0.51)		
	4:1	0.47 (0.70)	0.47 (0.71)	0.50 (0.25)	0.33 (0.73)	0.267	
	Total	0.44 (0.68)	0.39 (0.64)	0.47 (0.31)	0.29 (0.62)		
**Ab pain interference**	1:1	0.12 (0.39)	0.15 (0.53)	0.20 (0.47)	0.17 (0.61)		
	4:1	0.11 (0.39)	0.23 (0.66)	0.25 (0.55)	0.21 (0.49)	0.685	
	Total	0.12 (0.39)	0.19 (0.60)	0.22 (0.51)	0.19 (0.55)		
**Faecal incontinence frequency**	1:1	0.10 (0.30)	0.12 (0.46)	0.17 (0.52)	0.03 (0.18)		
	4:1	0.07 (0.26)	0.10 (0.37)	0.13 (0.48)	0.28 (0.65)	0.785	
	Total	0.08 (0.28)	0.11 (0.42)	0.15 (0.50)	0.16 (0.49)		
**Faecal incontinence interference**	1:1	0.04 (0.21)	0.08 (0.27)	0.05 (0.23)	0.17 (0.54)		
	4:1	0.02 (0.15)	0.07 (0.34)	0.08 (0.28)	0.17 (0.47)	0.925	
	Total	0.03 (0.18)	0.07 (0.31)	0.07 (0.25)	0.17 (0.50)		
**SOB severity**	1:1	0.22 (0.53)	0.15 (0.43)	0.20 (0.47)	0.25 (0.51)		
	4:1	0.52 (0.83)	0.52 (0.93)	0.50 (0.77)	0.32 (0.66)	0.012	4:1
	Total	0.37 (0.71)	0.34 (0.74)	0.35 (0.66)	0.28 (0.59)		
**SOB interference**	1:1	0.19 (0.55)	0.15 (0.43)	0.15 (0.44)	0.10 (0.41)		
	4:1	0.42 (0.83)	0.35 (0.66)	0.33 (0.79)	0.28 (0.65)	0.037	4:1
	Total	0.31 (0.71)	0.25 (0.56)	0.24 (0.65)	0.19 (0.55)		
**Cough severity**	1:1	0.21 (0.47)	0.20 (0.46)	0.23 (0.55)	0.14 (0.44)		
	4:1	0.28 (0.59)	0.30 (0.60)	0.19 (0.46)	0.32 (0.86)	0.494	
	Total	0.25 (0.53)	0.25 (0.54)	0.21 (0.50)	0.23 (0.68)		
**Cough interference**	1:1	0.09 (0.30)	0.13 (0.54)	0.09 (0.29)	0.00 (0.00)		
	4:1	0.11 (0.50)	0.13 (0.34)	0.13 (0.35)	0.32 (0.86)	0.237	
	Total	0.10 (0.41)	0.13 (0.44)	0.11 (0.32)	0.16 (0.62)		
**Wheezing**	1:1	0.12 (0.39)	0.05 (0.22)	0.00 (0.00)	0.03 (0.18)		
	4:1	0.11 (0.39)	0.05 (0.22)	0.03 (0.17)	0.14 (0.52)	0.570	
	Total	0.12 (0.39)	0.05 (0.22)	0.01 (0.12)	0.08 (0.39)		
**Swelling frequency**	1:1	0.24 (0.62)	0.26 (0.64)	0.24 (0.61)	0.17 (0.39)		
	4:1	0.64 (1.07)	0.51 (1.09)	0.52 (1.05)	0.23 (0.51)	0.065	
	Total	0.44 (0.90)	0.38 (0.90)	0.39 (0.87)	0.20 (0.45)		

**Swelling severity**	1:1	0.19 (0.55)	0.23 (0.54)	0.17 (0.45)	0.17 (0.47)		
	4:1	0.47 (0.86)	0.40 (0.77)	0.33 (0.67)	0.17 (0.39)	0.122	
	Total	0.33 (0.73)	0.32 (0.67)	0.25 (0.58)	0.17 (0.43)		
**Swelling interference**	1:1	0.07 (0.46)	0.05 (0.22)	0.05 (0.23)	0.07 (0.05)		
	4:1	0.33 (0.12)	0.17 (0.54)	0.50 (1.74)	0.07 (0.04)	0.038	
	Total	0.20 (0.07)	0.11 (0.42)	0.28 (1.27)	0.07 (0.03)		
**Heart palpitations frequency**	1:1	0.12 (0.45)	0.26 (0.60)	0.32 (0.68)	0.32 (0.61)		
	4:1	0.28 (0.67)	0.20 (0.51)	0.22 (0.54)	0.21 (0.56)	0.956	
	Total	0.20 (0.57)	0.23 (0.55)	0.27 (0.61)	0.26 (0.58)		
**Heart palpitations severity**	1:1	0.07 (0.26)	0.15 (0.36)	0.32 (0.57)	0.25 (0.51)		
	4:1	0.21 (0.08)	0.25 (0.63)	0.27 (0.51)	0.14 (0.35)	0.626	
	Total	0.14 (0.04)	0.20 (0.51)	0.28 (0.54)	0.19 (0.44)		
**Rash**	1:1	0.07 (0.26)	0.07 (0.27)	0.05 (0.23)	0.10 (0.31)		
	4:1	0.04 (0.21)	0.15 (0.36)	0.19 (0.40)	0.14 (0.35)	0.324	
	Total	0.06 (0.23)	0.11 (0.32)	0.12 (0.33)	0.12 (0.33)		
**Skin dryness**	1:1	0.39 (0.70)	0.42 (0.72)	0.44 (0.70)	0.32 (0.66)		
	4:1	0.69 (0.86)	0.72 (0.98)	0.66 (0.89)	0.71 (0.89)	0.034	4:1
	Total	0.54 (0.80)	0.57 (0.87)	0.55 (0.81)	0.51 (0.80)		
**Acne**	1:1	0.21 (0.52)	0.02 (0.16)	0.05 (0.23)	0.14 (0.53)		
	4:1	0.14 (0.35)	0.10 (0.30)	0.22 (0.63)	0.17 (0.47)	0.442	
	Total	0.18 (0.44)	0.06 (0.24)	0.14 (0.49)	0.16 (0.50)		
**Hair loss**	1:1	0.41 (0.77)	0.13 (0.34)	0.23 (0.60)	0.25 (0.58)		
	4:1	0.35 (0.75)	0.17 (0.54)	0.36 (0.76)	0.32 (0.81)	0.621	
	Total	0.38 (0.76)	0.15 (0.45)	0.30 (0.68)	0.28 (0.70)		
**Itching**	1:1	0.31 (0.72)	0.26 (0.55)	0.32 (0.53)	0.32 (0.72)		
	4:1	0.47 (0.86)	0.53 (0.78)	0.50 (0.84)	0.60 (0.78)	0.089	
	Total	0.39 (0.79)	0.40 (0.69)	0.41 (0.71)	0.46 (0.76)		
**Hives**	1:1	0.07 (0.26)	0.02 (0.16)	0.08 (0.28)	0.07 (0.26)		
	4:1	0.07 (0.26)	0.15 (0.36)	0.11 (0.31)	0.10 (0.31)	0.275	
	Total	0.07 (0.26)	0.08 (0.28)	0.10 (0.30)	0.08 (0.28)		
**Hand foot syndrome**	1:1	0.07 (0.34)	0.02 (0.16)	0.02 (0.17)	0.10 (0.31)		
	4:1	0.04 (0.21)	0.02 (0.15)	0.02 (0.16)	0.14 (0.44)	0.984	
	Total	0.06 (0.28)	0.02 (0.15)	0.02 (0.16)	0.12 (0.38)		
**Nail loss**	1:1	0.00 (0.00)	0.02 (0.16)	0.02 (0.17)	0.00 (0.00)		
	4:1	0.04 (0.03)	0.02 (0.15)	0.00 (0.00)	0.03 (0.18)	0.427	

	Total	0.02 (0.01)	0.02 (0.15)	0.01 (0.11)	0.01 (0.13)		
**Nail ridging**	1:1	0.12 (0.33)	0.10 (0.31)	0.11 (0.32)	0.10 (0.31)		
	4:1	0.11 (0.32)	0.10 (0.30)	0.08 (0.28)	0.21 (0.41)	0.908	
	Total	0.12 (0.32)	0.10 (0.30)	0.10 (0.30)	0.16 (0.37)		
**Nail dis-colouration**	1:1	0.02 (0.15)	0.00 (0.00)	0.05 (0.23)	0.10 (0.31)		
	4:1	0.07 (0.26)	0.05 (0.22)	0.11 (0.31)	0.10 (0.31)	0.300	
	Total	0.04 (0.21)	0.02 (0.15)	0.08 (0.28)	0.10 (0.31)		
**Phyto-sensitivity**	1:1	0.14 (0.35)	0.02 (0.16)	0.02 (0.17)	0.07 (0.26)		
	4:1	0.04 (0.21)	0.03 (0.22)	0.05 (0.23)	0.03 (0.18)	0.404	
	Total	0.09 (0.29)	0.02 (0.19)	0.04 (0.20)	0.05 (0.22)		
**Bed sores**	1:1	0.02 (0.15)	0.02 (0.16)	0.02 (0.02)	0.00 (0.00)		
	4:1	0.00 (0.00)	0.00 (0.00)	0.00 (0.00)	0.00 (0.00)	0.230	
	Total	0.01 (0.10)	0.01 (0.11)	0.01 (0.01)	0.00 (0.00)		
**Radiation skin reactions**	1:1	4.31 (1.57)	0.35 (1.22)	4.70 (1.19)	4.57 (1.34)		
	4:1	4.21 (1.68)	0.00 (0.00)	4.58 (1.40)	4.39 (1.59)	0.417	
	Total	4.26 (0.62)	0.16 (0.86)	4.64 (1.29)	4.48 (1.46)		
**Skin darkening**	1:1	0.07 (0.26)	0.02 (0.16)	0.03 (0.17)	0.12 (0.33)		
	4:1	0.04 (0.21)	0.02 (0.15)	0.08 (0.36)	0.03 (0.19)	0.766	
	Total	0.06 (0.24)	0.02 (0.15)	0.05 (0.29)	0.07 (0.27)		
**Stretch marks**	1:1	0.02 (0.15)	0.02 (0.16)	0.06 (0.24)	0.07 (0.26)		
	4:1	0.02 (0.15)	0.05 (0.22)	0.05 (0.33)	0.03 (0.18)	1.000	
	Total	0.02 (0.15)	0.03 (0.19)	0.05 (0.29)	0.05 (0.22)		
**Numbness and tingling severity**	1:1	0.39 (0.73)	0.28 (0.68)	0.36 (0.89)	0.28 (0.65)		
	4:1	0.76 (0.95)	0.55 (0.74)	0.55 (0.77)	0.17 (0.39)	0.076	
	Total	0.57 (0.87)	0.41 (0.72)	0.46 (0.83)	0.23 (0.53)		
**Numbness and tingling interference**	1:1	0.26 (0.67)	0.25 (0.63)	0.28 (0.88)	0.21 (0.62)		
	4:1	0.33 (0.78)	0.37 (0.70)	0.33 (0.58)	0.07 (0.26)	0.637	
	Total	0.30 (0.72)	0.31 (0.67)	0.30 (0.73)	0.14 (0.48)		
**Dizziness severity**	1:1	0.51 (0.63)	0.43 (0.71)	0.48 (0.71)	0.46 (0.83)		
	4:1	0.76 (0.95)	0.80 (0.91)	0.75 (0.99)	0.46 (0.74)	0.071	4:1
	Total	0.63 (0.82)	0.62 (0.83)	0.62 (0.87)	0.46 (0.78)		
**Dizziness interference**	1:1	0.39 (0.58)	0.28 (0.60)	0.42 (0.75)	0.50 (0.17)		
	4:1	0.64 (0.98)	0.42 (0.81)	0.58 (0.87)	0.37 (0.13)	0.218	
	Total	0.51 (0.81)	0.35 (0.71)	0.50 (0.81)	0.43 (0.10)		
**Blurred vision severity**	1:1	0.48 (0.77)	0.51 (0.96)	0.45 (0.97)	0.40 (0.93)		
	4:1	0.66 (0.90)	0.47 (0.84)	0.58 (0.87)	0.55 (0.80)	0.452	

	Total	0.57 (0.84)	0.49 (0.90)	0.52 (0.91)	0.48 (0.86)		
**Blurred vision interference**	1:1	0.32 (0.69)	0.41 (0.93)	0.36 (0.82)	0.39 (0.91)		
	4:1	0.54 (0.99)	0.40 (0.70)	0.58 (0.90)	0.42 (0.83)	0.292	
	Total	0.43 (0.86)	0.40 (0.82)	0.47 (0.86)	0.41 (0.86)		
**Flashing light severity**	1:1	0.04 (0.21)	0.07 (0.26)	0.18 (0.58)	0.28 (0.53)		
	4:1	0.19 (0.45)	0.15 (0.36)	0.25 (0.50)	0.14 (0.35)	0.320	
	Total	0.12 (0.36)	0.11 (0.31)	0.21 (0.53)	0.21 (0.45)		
**Visual floaters**	1:1	0.12 (0.33)	0.13 (0.34)	0.18 (0.39)	0.28 (0.46)		
	4:1	0.38 (0.49)	0.35 (0.53)	0.30 (0.52)	0.21 (0.41)	0.037	1:1
	Total	0.25 (0.43)	0.24 (0.46)	0.24 (0.46)	0.25 (0.43)		
**Watery eyes severity**	1:1	0.17 (0.44)	0.10 (0.30)	0.42 (0.66)	0.25 (0.52)		
	4:1	0.28 (0.67)	0.25 (0.58)	0.27 (0.56)	0.10 (0.31)	0.700	
	Total	0.22 (0.57)	0.17 (0.47)	0.34 (0.61)	0.18 (0.43)		
**Watery eyes interference**	1:1	0.12 (0.39)	0.07 (0.26)	0.09 (0.29)	0.18 (0.39)		
	4:1	0.21 (0.64)	0.12 (0.33)	0.13 (0.35)	0.00 (0.00)	0.660	
	Total	0.16 (0.53)	0.10 (0.30)	0.11 (0.32)	0.09 (0.29)		
**Ringing in the ears**	1:1	0.34 (0.65)	0.23 (0.53)	0.30 (0.58)	0.46 (0.74)		
	4:1	0.45 (0.77)	0.32 (0.65)	0.30 (0.57)	0.25 (0.51)	0.581	
	Total	0.39 (0.71)	0.27 (0.59)	0.30 (0.57)	0.35 (0.64)		
**Concentration severity**	1:1	0.85 (0.90)	0.71 (0.85)	0.90 (1.02)	0.71 (0.89)		
	4:1	1.26 (1.08)	1.07 (0.82)	1.05 (1.04)	1.00 (0.94)	0.033	4:1
	Total	1.06 (1.01)	0.89 (0.85)	0.98 (1.02)	0.85 (0.92)		
**Concentration frequency**	1:1	0.68 (0.81)	0.50 (0.79)	0.81 (1.13)	0.75 (0.96)		
	4:1	1.26 (1.26)	1.00 (0.96)	0.94 (0.95)	0.75 (0.92)	0.027	4:1
	Total	0.97 (1.10)	0.75 (0.91)	0.88 (1.03)	0.75 (0.93)		
**Memory severity**	1:1	0.87 (0.82)	0.75 (0.72)	0.96 (0.99)	0.89 (0.95)		
	4:1	1.11 (1.19)	1.05 (0.95)	1.08 (1.02)	1.07 (1.01)	0.115	
	Total	1.00 (1.03)	0.90 (0.86)	1.02 (1.00)	0.98 (0.98)		
**Memory interference**	1:1	0.73 (0.74)	0.48 (0.85)	0.78 (0.94)	0.78 (0.99)		
	4:1	1.02 (1.07)	0.87 (0.85)	0.77 (0.84)	0.71 (0.93)	0.175	
	Total	0.87 (0.92)	0.68 (0.87)	0.77 (0.88)	0.75 (0.95)		
**General pain frequency**	1:1	0.87 (1.03)	0.43 (0.68)	0.53 (0.71)	0.66 (0.87)		
	4:1	0.97 (0.09)	0.82 (1.08)	0.82 (1.09)	0.92 (1.35)	0.192	
	Total	0.93 (1.05)	0.63 (0.93)	0.68 (0.94)	0.80 (1.14)		
**General pain severity**	1:1	0.70 (0.87)	0.33 (0.57)	0.69 (1.04)	0.46 (0.57)		
	4:1	0.78 (0.97)	0.62 (0.83)	0.58 (0.80)	0.53 (0.92)	0.511	
	Total	0.74 (0.92)	0.48 (0.73)	0.63 (0.92)	0.50 (0.76)		

**General pain interference**	1:1	0.48 (0.89)	0.23 (0.58)	0.30 (0.63)	0.32 (0.47)		
	4:1	0.68 (0.96)	0.52 (0.81)	0.61 (0.96)	0.42 (0.92)	0.107	
	Total	0.58 (0.92)	0.38 (0.72)	0.46 (0.83)	0.37 (0.72)		
**Headache frequency**	1:1	0.92 (1.05)	0.51 (0.68)	0.81 (0.99)	0.64 (0.73)		
	4:1	1.07 (1.11)	0.85 (1.02)	0.75 (1.07)	0.67 (0.90)	0.258	
	Total	1.00 (1.08)	0.68 (0.88)	0.77 (1.03)	0.66 (0.81)		
**Headache severity**	1:1	0.80 (0.92)	0.50 (0.78)	0.66 (0.88)	0.53 (0.57)		
	4:1	0.80 (0.89)	0.66 (0.92)	0.61 (0.83)	0.53 (0.69)	0.717	
	Total	0.80 (0.90)	0.58 (0.86)	0.63 (0.85)	0.53 (0.63)		
**Headache interference**	1:1	0.55 (0.87)	0.25 (0.55)	0.32 (0.58)	0.42 (0.63)		
	4:1	0.54 (0.83)	0.39 (0.67)	0.52 (0.86)	0.25 (0.58)	0.593	
	Total	0.54 (0.84)	0.32 (0.62)	0.42 (0.73)	0.33 (0.61)		
**Muscle pain frequency**	1:1	0.85 (1.09)	0.86 (1.25)	0.54 (0.79)	0.60 (0.78)		
	4:1	1.14 (1.33)	0.89 (1.07)	0.88 (1.05)	1.03 (1.31)	0.161	
	Total	1.00 (0.22)	0.88 (1.15)	0.72 (0.94)	0.81 (1.09)		
**Muscle pain Severity**	1:1	0.60 (0.89)	0.58 (0.73)	0.50 (0.61)	0.44 (0.50)		
	4:1	0.85 (1.02)	0.66 (0.86)	0.68 (0.83)	0.78 (0.91)	0.121	
	Total	0.73 (0.96)	0.62 (0.80)	0.59 (0.73)	0.61 (0.75)		
**Muscle pain interference**	1:1	0.41 (0.83)	0.29 (0.57)	0.35 (0.59)	0.25 (0.44		
	4:1	0.83 (1.08)	0.53 (0.96)	0.51 (0.75)	0.57 (0.92)	0.019	4:1
	Total	0.62 (0.98)	0.42 (0.80)	0.43 (0.67)	0.41 (0.73)		
**Joint pain frequency**	1:1	0.67 (0.94)	0.56 (1.14)	0.61 (1.01)	0.57 (0.92)		
	4:1	0.88 (1.25)	0.51 (0.88)	0.71 (1.20)	0.75 (0.96)	0.460	
	Total	0.78 (1.11)	0.53 (1.01)	0.66 (1.10)	0.66 (0.93)		
**Joint pain severity**	1:1	0.48 (0.74)	0.43 (0.68)	0.52 (0.78)	0.35 (0.48)		
	4:1	0.73 (0.98)	0.43 (0.82)	0.48 (0.81)	0.64 (0.86)	0.286	
	Total	0.61 (0.88)	0.43 (0.75)	0.50 (0.79)	0.50 (0.71)		
**Joint pain interference**	1:1	0.43 (0.97)	0.24 (0.54)	0.48 (0.75)	0.21 (0.41)		
	4:1	0.69 (1.17)	0.35 (0.77)	0.45 (0.85)	0.46 (0.79)	0203	
	Total	0.56 (1.08)	0.30 (0.67)	0.47 (0.80)	0.33 (0.64)		
**Insomnia severity**	1:1	0.68 (1.08)	0.37 (0.72)	0.32 (0.53)	0.35 (0.62)		
	4:1	1.45 (1.31)	0.64 (0.87)	0.51 (0.78)	0.46 (0.69)	0.011	4:1
	Total	1.07 (1.25)	0.51 (0.80)	0.42 (0.67)	0.41 (0.65)		
**Insomnia frequency**	1:1	0.58 (0.86)	0.37 (0.68)	0.41 (0.65)	0.28 (0.53)		
	4:1	1.11 (1.17)	0.56 (0.88)	0.45 (0.78)	0.42 (0.69)	0.076	4:1

	Total	0.85 (1.06)	0.47 (0.79)	0.43 (0.71)	0.35 (0.61)		
**Fatigue severity**	1:1	1.35 (1.02)	1.24 (1.21)	1.32 (1.14)	1.17 (0.94)		
	4:1	1.78 (1.27)	1.61 (1.06)	1.60 (1.21)	1.40 (1.15)	0.051	4:1
	Total	1.57 (1.57)	1.43 (1.14)	1.46 (1.18)	1.29 (1.04)		
**Fatigue interference**	1:1	1.17 (0.97)	1.02 (1.25)	1.23 (1.18)	0.96 (0.92)		
	4:1	1.52 (1.29)	1.23 (1.13)	1.51 (1.24)	1.14 (1.23)	0.146	
	Total	1.34 (1.15)	1.13 (1.19)	1.37 (1.21)	1.05 (1.07)		
**Anxiety frequency**	1:1	1.12 (1.17)	0.86 (1.00)	1.06 (1.05)	1.07 (1.05)		
	4:1	1.33 (1.24)	0.82 (0.84)	1.02 (0.98)	0.96 (0.87)	0.871	
	Total	1.27 (1.20)	0.84 (0.91)	1.04 (1.01)	1.01 (0.96)		
**Anxiety severity**	1:1	0.73 (0.86)	0.52 (0.68)	0.79 (0.88)	0.78 (0.83)		
	4:1	1.04 (1.03)	0.60 (0.70)	0.94 (0.95)	0.78 (0.78)	0.240	
	Total	0.89 (0.96)	0.56 (0.69)	0.86 (0.91)	0.78 (0.80)		
**Anxiety interference**	1:1	0.53 (0.77)	0.28 (0.51)	0.38 (0.69)	0.35 (0.55)		
	4:1	0.76 (1.00)	0.32 (0.57)	0.48 (0.65)	0.35 (0.62)	0.289	
	Total	0.65 (0.90)	0.30 (0.54)	0.43 (0.67)	0.35 (0.58)		
**Discouraged frequency**	1:1	0.82 (0.97)	0.57 (0.91)	0.67 (0.91)	0.82 (0.86)		
	4:1	0.88 (1.06)	0.37 (0.54)	0.54 (0.78)	0.53 (0.74)	0.488	
	Total	0.85 (1.01)	0.47 (0.75)	0.60 (0.84)	0.67 (0.81)		
**Discouraged severity**	1:1	0.65 (0.79)	0.32 (0.62)	0.52 (0.82)	0.66 (0.73)		
	4:1	0.66 (0.90)	0.32 (0.47)	0.54 (0.81)	0.46 (0.63)	0.892	
	Total	0.66 (0.84)	0.32 (0.54)	0.53 (0.81)	0.56 (0.68)		
**Discouraged interference**	1:1	0.43 (0.70)	0.21 (0.58)	0.54 (0.83)	0.37 (0.56)		
	4:1	0.57 (0.85)	0.32 (0.61)	0.37 (0.59)	0.39 (0.68)	0.619	
	Total	0.50 (0.78)	0.27 (0.59)	0.45 (0.72)	0.38 (0.62)		
**Sad frequency**	1:1	1.29 (1.10)	0.97 (0.94)	2.26 (7.08)	1.07 (0.93)		
	4:1	1.14 (0.92)	0.94 (0.82)	1.00 (0.93)	0.71 (0.80)	0.237	
	Total	1.21 (1.01)	0.96 (0.88)	1.62 (5.02)	0.89 (0.88)		
**Sad severity**	1:1	1.02 (0.96)	0.70 (0.81)	0.94 (0.88)	0.82 (0.72)		
	4:1	0.97 (0.84)	0.82 (0.84)	0.74 (0.78)	0.64 (0.73)	0.799	
	Total	1.00 (0.89)	0.76 (0.82)	0.84 (0.83)	0.73 (0.72)		
**Sad interference**	1:1	0.51 (0.74)	0.43 (0.76)	0.50 (0.82)	0.50 (0.63)		
	4:1	0.61 (0.69)	0.40 (0.74)	0.38 (0.60)	0.39 (0.73)	0.903	
	Total	0.56 (0.71)	0.41 (0.74)	0.44 (0.72)	0.44 (0.68)		
**Bruising**	1:1	0.17 (0.38)	0.13 (0.34)	0.17 (0.38)	0.17 (0.39)		
	4:1	0.35 (0.48)	0.17 (0.38)	0.34 (0.53)	0.25 (0.44)	0.064	4:1

	Total	0.26 (0.44)	0.15 (0.36)	0.26 (0.47)	0.21 (0.41)		
**Chills frequency**	1:1	0.29 (0.67)	0.18 (0.60)	0.50 (0.96)	0.39 (0.78)		
	4:1	0.51 (0.84)	0.37 (0.83)	0.42 (0.69)	0.71 (1.04)	0.187	
	Total	0.40 (0.76)	0.28 (0.73)	0.46 (0.83)	0.55 (0.93)		
**Chills severity**	1:1	0.17 (0.38)	0.08 (0.27)	0.38 (0.73)	0.42 (0.69)		
	4:1	0.36 (0.62)	0.22 (0.57)	0.26 (0.51)	0.67 (0.90)	0.189	
	Total	0.26 (0.52)	0.15 (0.46)	0.32 (0.63)	0.55 (0.80)		
**Sweating frequency**	1:1	0.43 (0.80)	0.28 (0.73)	0.33 (0.54)	0.14 (0.44)		
	4:1	0.52 (1.03)	0.25 (0.63)	0.28 (0.62)	0.50 (0.83)	0.472	
	Total	0.48 (0.92)	0.27 (0.68)	0.30 (0.57)	0.32 (0.69)		
**Sweating severity**	1:1	0.29 (0.60)	0.20 (0.52)	0.34 (0.54)	0.07 (0.26)		
	4:1	0.41 (0.86)	0.25 (0.63)	0.25 (0.64)	0.35 (0.62)	0.470	
	Total	0.35 (0.74)	0.22 (0.57)	0.29 (0.59)	0.21 (0.49)		
**Decreased sweating**	1:1	0.00 (0.00)	0.00 (0.00)	0.03 (0.17)	0.00 (0.00)		
	4:1	0.07 (0.34)	0.12 (0.64)	0.02 (0.16)	0.10 (0.56)	0.069	4:1
	Total	0.03 (0.24)	0.06 (0.46)	0.02 (0.17)	0.05 (0.40)		
**Hot flashes frequency**	1:1	0.17 (0.54)	0.20 (0.61)	0.18 (0.52)	0.17 (0.61)		
	4:1	0.47 (0.89)	0.40 (0.81)	0.22 (0.63)	0.39 (0.78)	0.171	
	Total	0.32 (0.75)	0.30 (0.72)	0.20 (0.58)	0.28 (0.70)		
**Hot flashes interference**	1:1	0.17 (0.62)	0.13 (0.41)	0.12 (0.41)	0.14 (0.45)		
	4:1	0.33 (0.65)	0.25 (0.58)	0.22 (0.63)	0.25 (0.51)	0.334	
	Total	0.25 (0.64)	0.19 (0.51)	0.17 (0.54)	0.20 (0.48)		
**Nose bleeds frequency**	1:1	0.02 (0.15)	0.02 (0.16)	0.00 (0.00)	0.03 (0.18)		
	4:1	0.14 (0.35)	0.12 (0.40)	0.13 (0.42)	0.03 (0.18)	0.049	4:1
	Total	0.08 (0.27)	0.07 (0.31)	0.07 (0.31)	0.03 (0.18)		
**Nose bleeds severity**	1:1	0.02 (0.15)	0.02 (0.16)	0.00 (0.00)	0.03 (0.18)		
	4:1	0.11 (0.32)	0.07 (0.26)	0.11 (0.32)	0.03 (0.18)	0.108	
	Total	0.07 (0.26)	0.05 (0.22)	0.05 (0.23)	0.03 (0.18)		
**Pain and swelling**	1:1	0.00 (0.00)	0.43 (1.04)	0.66 (1.19)	0.82 (1.30)		
	4:1	0.00 (0.00)	0.45 (1.03)	0.55 (1.05)	0.71 (1.24)	0.755	
	Total	0.00 (0.00)	0.44 (1.03)	0.60 (1.11)	0.76 (1.26)		
**Body odour**	1:1	0.26 (0.59)	0.25 (0.59)	0.39 (0.70)	0.25 (0.51)		
	4:1	0.23 (0.48)	0.45 (0.67)	0.25 (0.55)	0.25 (0.44)	0.740	
	Total	0.25 (0.53)	0.35 (0.64)	0.31 (0.62)	0.25 (0.47)		
